# Differential analysis of N-glycoproteome between hepatocellular carcinoma and normal human liver tissues by combination of multiple protease digestion and solid phase based labeling

**DOI:** 10.1186/1559-0275-11-26

**Published:** 2014-07-01

**Authors:** Zhen Sun, Deguang Sun, Fangjun Wang, Kai Cheng, Zhang Zhang, Bo Xu, Mingliang Ye, Liming Wang, Hanfa Zou

**Affiliations:** 1 Key Lab of Separation Sciences for Analytical Chemistry, National Chromatographic Research and Analysis Center, Dalian Institute of Chemical Physics, Chinese Academy of Sciences, Dalian 116023, China; 2 The Second Affiliated Hospital of Dalian Medical University, Dalian 116027, China

**Keywords:** N-glycoproteome, N-glycosite, Multiple protease digestion, Quantitative analysis

## Abstract

**Background:**

Dysregulation of glycoproteins is closely related with many diseases. Quantitative proteomics methods are powerful tools for the detection of glycoprotein alterations. However, in almost all quantitative glycoproteomics studies, trypsin is used as the only protease to digest proteins. This conventional method is unable to quantify N-glycosites in very short or long tryptic peptides and so comprehensive glycoproteomics analysis cannot be achieved.

**Methods:**

In this study, a comprehensive analysis of the difference of N-glycoproteome between hepatocellular carcinoma (HCC) and normal human liver tissues was performed by an integrated workflow combining the multiple protease digestion and solid phase based labeling. The quantified N-glycoproteins were analyzed by GoMiner to obtain a comparative view of cellular component, biological process and molecular function.

**Results/conclusions:**

An integrated workflow was developed which enabled the processes of glycoprotein coupling, protease digestion and stable isotope labeling to be performed in one reaction vessel. This workflow was firstly evaluated by analyzing two aliquots of the same protein extract from normal human liver tissue. It was demonstrated that the multiple protease digestion improved the glycoproteome coverage and the quantification accuracy. This workflow was further applied to the differential analysis of N-glycoproteome of normal human liver tissue and that with hepatocellular carcinoma. A total of 2,329 N-glycosites on 1,052 N-glycoproteins were quantified. Among them, 858 N-glycosites were quantified from more than one digestion strategy with over 99% confidence and 1,104 N-glycosites were quantified from only one digestion strategy with over 95% confidence. By comparing the GoMiner results of the N-glycoproteins with and without significant changes, the percentage of membrane and secreted proteins and their featured biological processes were found to be significant different revealing that protein glycosylation may play the vital role in the development of HCC.

## Background

Protein glycosylation, one of the most important post-translational modifications of proteins, plays a pivotal role in many biological pathways including cell-cell signaling, ion transport, protein stability, vesicle trafficking and so on [1,2]. Aberrant glycosylation has been proved to be associated with disease progression, carcinogenesis and immunity [3-5]. Currently, many glycosylated proteins are approved to be clinical biomarkers, e.g., prostate-specific antigen (PSA) in prostate cancer, cancer antigen (CA) 125 in ovarian cancer, α-fetoprotein (AFP) in HCC, and HER2/neu in breast cancer. Therefore, quantitative analysis of disease-associated alteration in protein glycosylation can help in prognosis, diagnosis and surveillance after surgery.

Several methods have been developed for glycoproteomics analysis, e.g. hydrazide chemistry [6], hydrophilic interaction chromatography (HILIC) [7], lectin affinity chromatography [8], boronic acid chromatography [9], titanium dioxide [10], etc. Considering the facts that hydrazide chemistry can isolate N-glycopeptides with specificity of more than 90% [11] and is compatible with stable isotope labeling, we previously developed a solid phase based labeling approach by integration of N-glycopeptide enrichment and the fast and simple dimethyl labeling derivatization on hydrazide resins for relative quantification of protein glycosylation. It was found that this approach has higher enrichment recovery and detection sensitivity than the dimethyl labeling approach conventionally performed in solution [12].

Liver is the largest visceral organ which is necessary for survival in human body. It involves in a wide range of biological processes, including detoxification, protein synthesis, and production of biochemical necessary for digestion. Liver cancer is the third most common cause of cancer death after lung cancer and stomach cancer [13,14]. Newly developed proteomic techniques have been applied to deeply analyze the proteins and their modifications in human liver tissue. Song et al. conducted a large-scale phosphorylation analysis of human liver and experimentally identified 9,719 p-sites in 2,998 proteins [15]. Chen et al. identified 939 N-glycosylation sites in 523 N-glycosylated proteins by combining multiple protease digestion and hydrazide chemistry for human liver N-glycoproteome analysis [16]. Furthermore, several studies on comparative analyses of HCC and normal human liver tissues were carried out to screen potential disease-specific biomarkers. In our lab, Wang et al. have done quantitative analysis of HCC and normal human liver tissues in ~30 h by using a fully automated system which quantified ~1,000 proteins [17]. Moreover, the difference in the phosphoproteomes of HCC and normal human liver tissues was also investigated by Song et al. [18], with over 1,800 phosphopeptides corresponding to ~1,000 phosphoproteins reliably quantified in only 42 h using a pseudo triplex labeling system. Nevertheless, the differences in the N-glycoproteome of HCC and normal human liver tissues was still of great importance to be extensively studied.

Using multiple proteases with complementary cleavage specificities for digestion can efficiently improve protein identifications and proteome sequence coverage. This method has already been applied to qualitative analysis of proteome, phosphoproteome, and glycoproteome [16,19-22]. However, no attempt was performed to quantitative analysis of proteome, phosphoproteome and glycoproteome. In this study, digestion with three different digestion strategies (trypsin, trypsin & Glu-C, and chymotrypsin) was combined with the solid phase based labeling approach to comparatively analyze the differential expression of N-glycoproteome between HCC and normal human liver tissues. A total of 2,329 N-glycosites matched with the motif N-X-S/T (X can be any amino acid except proline) on 1,052 N-glycoproteins were quantified by this strategy, which is the largest dataset of quantitative information between human HCC and normal liver tissues up to now.

## Results

### An integrated workflow incorporated with multiple protease digestion and solid phase based labeling

**Figure 1:**
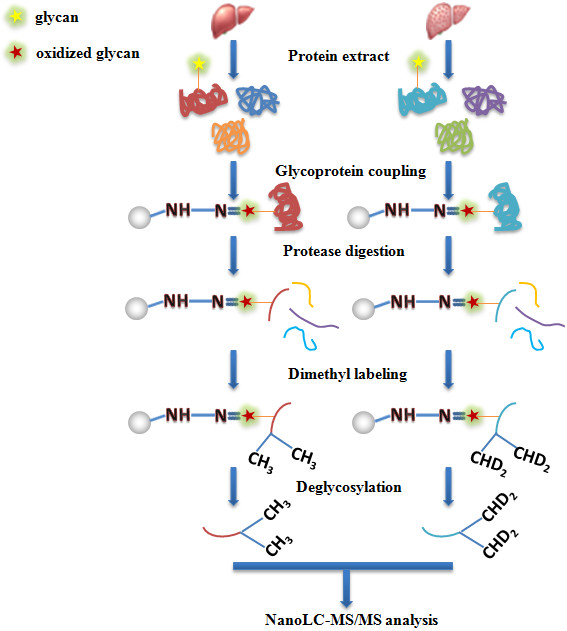
**The integrated workflow for high-throughput quantitative analysis of N-glycoproteome of human liver tissues.** Three digestion strategies by using proteases with different cleavage specificities were applied to digest the captured N-glycoproteins.

For a convenient and fast-processing workflow, all the processes including glycoprotein coupling, protease digestion and stable isotope labeling should be performed in one reaction vessel. As shown in Figure 1, the workflow developed in this study enabled the performing of all the above steps in one vessel. The extract of human liver tissues was oxidized by sodium periodate in a centrifugal tube, followed by adding hydrazide resins to the tube for the capturing of the oxidized glycoproteins. After washing, the resins with captured glycoproteins were still left in the tube. Protease was then added to the tube for the digestion of the captured glycoproteins on the hydrazide resins. After digestion, the non-glycosylated peptides were released from the resins and washed away. The remaining glycopeptides on the resins were left in the tube. Dimethyl labeling reagents were then added to the tube for stable isotope labeling of the glycopeptides on the resins. Finally, these labeled N-glycopeptides were released by deglycosylation with PNGase F. After centrifugation, the released light and heavy labeled deglycosylated peptides were collected and then pooled together for nanoLC-MS/MS analysis.

This workflow firstly benefits from the fact that the enrichment process was carried out on the protein level. As the glycoproteins are covalently captured on the hydrazide resins, the exchanges of buffers are extremely convenient. Since most of the membrane proteins are glycosylated, detergents with high concentrations (i.e. 4% SDS) were added in the homogenization buffer to facilitate the extract of membrane glycoproteins. It is challenging to remove these detergents in conventional approach, while in this workflow they are easily removed by washing the hydrazide resins with 80% ACN. This solid phase design also benefits the downstream sample processes. For example, it allows the labeling of glycopeptides on the hydrazide resins, i.e. solid phase based labeling of glycopeptides for quantitative glycoproteomics. This labeling method was previously proved to be accurate and has good enrichment recovery and high detection sensitivity [12].

Using multiple proteases for digestion is an essential approach to increase the sequence coverage for glycoproteome analysis and the confidence of quantification results of N-glycosites. In addition to trypsin, Glu-C and chymotrypsin were also applied to digest glycoproteins in this study. Trypsin cleavages specifically the C-terminus of basic residues (K and R), while Glu-C cuts the C-terminus of acid residues (D and E) and chymotrypsin cuts the C-terminus of hydrophobic residues (Y, W, F and L). As the other two proteases have complementary cleavage specificities to that of trypsin, it can be expected that many N-glycosites which cannot be identified by trypsin digestion are possibly to be identified by other protease digestions. For the quantification of N-glycosites, it is different with protein quantification in which the result can be obtained by averaging all the quantification results of different tryptic peptides from the parent protein. Nevertheless, applying multiple protease digestion may quantify different N-glycopeptides containing the same N-glycosite. Thus the accuracy for the quantification results of N-glycosites could be improved by averaging the ratios of different N-glycopeptides containing the same N-glycosites. Therefore, it can be expected that this integrated workflow incorporated with multiple protease digestion and solid phase based labeling can be applied to deeply inspect the N-glycosite abundance differences of tissue glycoproteomes.

### Evaluating the performance of the integrated workflow

**Figure 2:**
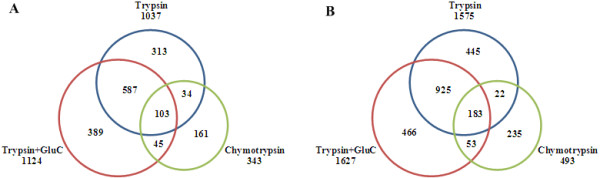
**The venn diagram showing the overlap of the quantified N-glycosites by using three different digestion strategies (trypsin, trypsin & Glu-C, chymotrypsin). (A)** evaluation experiment, **(B)** differential analysis experiment. For the evaluation experiment, two aliquots of the same protein extract from normal human liver tissue were light and heavy labeled to evaluate the performance of the integrated workflow. For the differential analysis experiment, the samples of normal and HCC human liver tissues were labeled with light and heavy dimethyl labels, respectively. One and three replicate 2D nanoLC-MS/MS runs of the labeled sample were carried out for the evaluation experiment and differential analysis experiment, respectively.

Firstly, the integrated workflow was evaluated by quantitative analysis of two identical samples, i.e. two aliquots of the same protein extract from normal human liver tissue. A total of 1,632 N-glycosites corresponding to 764 N-glycoproteins were successfully quantified by the three digestion strategies (Additional file 1: Table S1). Only one protease was used for trypsin digestion and chymotrypsin digestion. While for trypsin & Glu-C digestion, these two proteases were added together into the samples for digestion. This is because that Glu-C generated peptides are too big to be identified by MS [19,21] and the *in silico* sequence coverage for combined trypsin & Glu-C digests was proved to achieve the greatest coverage [20]. The trypsin digestion leaded to quantification of only 1,037 N-glycosites, while the total number of quantified N-glycosites reached 1,632 by using other two digestion strategies (Figure 2A). The number increased by 57.4% indicating the using of complementary proteases does improve the N-glycoproteome coverage significantly. Target/decoy search was performed in this study to control the confidence of peptide identifications. Among all the quantified N-glycosites, only 5 N-glycosites were quantified from decoy sequences. It was found the decoy identifications were only found in the results from one digestion strategy, while no decoy identification was observed for overlapped quantified N-glycosites. Clearly the N-glycosites quantified by more than one digestion strategy are more confident simply because these sites were quantified by multiple N-glycopeptides. Thus, the identification confidence of the 47.1% of the total identified N-glycosites was higher than that of others for they were observed in digestion results from more than one digestion strategy.

**Figure 3:**
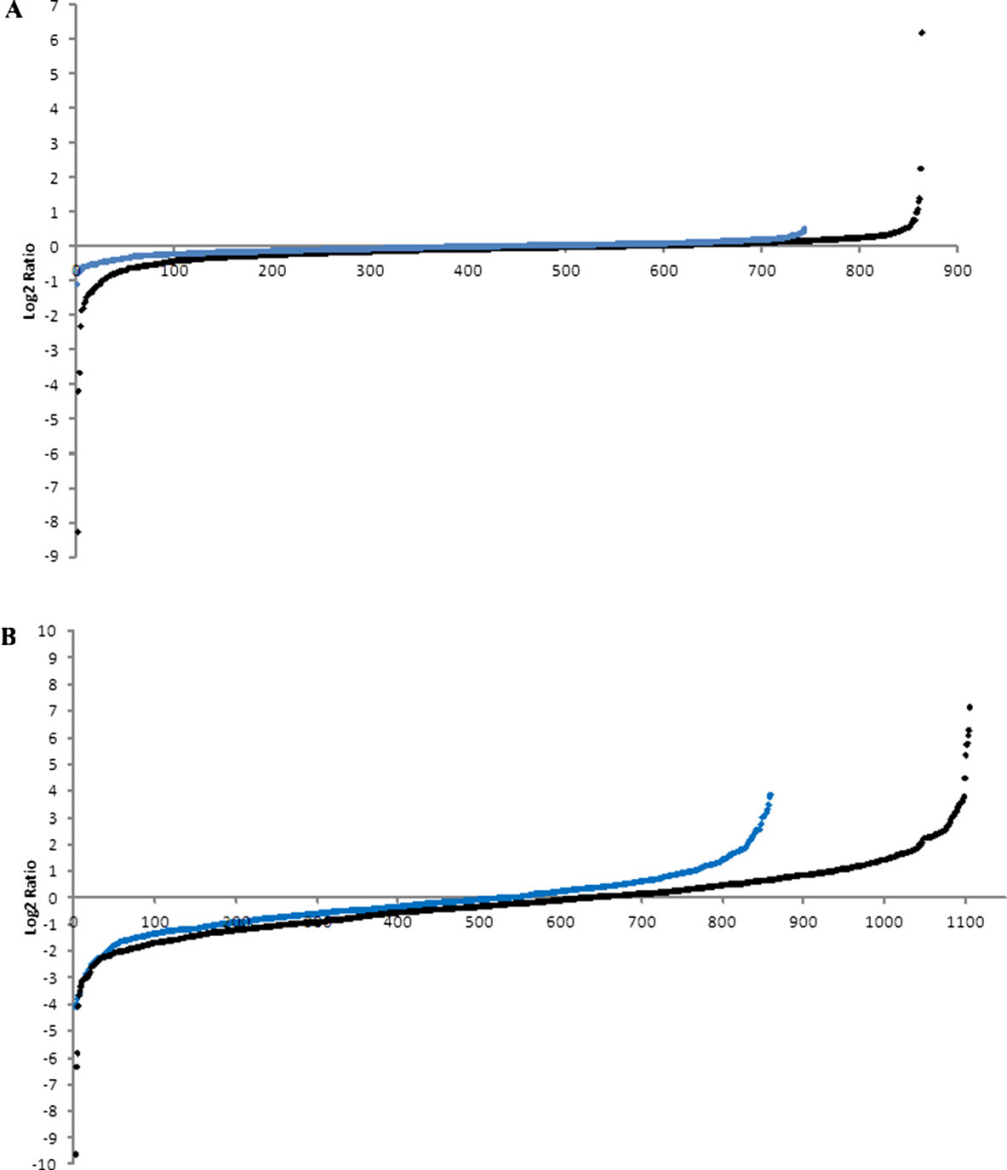
**The scatter diagram showing the log2 ratio distributions of N-glycosites quantified from more than one digestion strategy with RSD <50% (blue dot) and that from only one digestion strategy (black dot). (A)** evaluation experiment, **(B)** differential analysis experiment. The description for the two experiments was the same as in Figure 2.

Statistically, multiple measurements are essential to improve the analysis accuracy. However, only one measurement is done for conventional quantitative glycoproteomics analysis as majority of N-glycosites are quantified by a single glycopeptide. The main reason is that only one digestion strategy, e.g. trypsin digestion, was commonly used. While in this study some N-glycosites were quantified by several glycopeptides thanks to the using of multiple proteases. For example, the 13 amino acid sequence window of N-glycosite N119 on ERAP2 is KDIEITNATIQSE. This sites were quantified by three different glycopeptides, i.e. DIEITN*ATIQSEEDSR (N* indicates that asparagine was detected with deamidation) with heavy/light ratio 0.85 by trypsin digestion, ITN*ATIQSEEDSR with heavy/light ratio 1.03 by trypsin & Glu-C digestion, and IIIHSKDIEITN*ATIQSEEDSRY with heavy/light ratio 1.12 by chymotrypsin digestion. Thus this N-glycosite was quantified to be heavy/light ratio of 1.00 by averaging above three ratios. More importantly, RSD could be determined for multiple measurements. For above case, the RSD was determined to be 13.7%, indicating this site was reliably quantified. The total quantified N-glycosites were classified into two groups: 1) the N-glycosites quantified from more than one digestion strategy; 2) the N-glycosites quantified from only one digestion strategy. For the first group, RSD could be determined among the ratios obtained from different digestion strategies. The RSD values could be used to filter out the unreliable quantified results. The distribution of the number of quantified N-glycosites and the percentage of quantified ratio within the range of 0.5-2 across different RSD values are given in Additional file 2: Figure S1. In proteomics, the quantified ratios in the range of 0.5-2 are often considered as no significant change [17,23-25]. In this evaluation experiment, two identical samples were light and heavy labeled, the theoretical ratios are 1:1 and the ratios for all quantified sites should be in the range of 0.5-2. If the ratio beyond this range, it can be considered as inaccurate quantification. It can be seen from Additional file 2: Figure S1, only 1 of the 26 N-glycosites (3.8%) were quantified with heavy/light ratio in the range of 0.5-2 for N-glycosites with RSD > =50%. This means that the quantified ratios with RSD > =50% are not accurate. The percentage of quantified N-glycosites within this ratio range increased to 100% when the RSD value fell below 50%, and kept on 100% when the RSD value continued to decrease to 20%. But the number of quantified N-glycosites filtered with RSD value decreased significantly along with the decrease of the RSD value. So we adopted the criterion of RSD <50% to exclude the inaccurate quantification and make relatively more peptides meeting the criterion for research with no sacrifice of the confidence of quantification. After filtering with RSD <50%, about 99.9% of the quantified N-glycosites were located in the ratio range of 0.5-2 with only one exception for this group (Figure 3A). While for the second group, among the 863 quantified N-glycosites, as many as 32 N-glycosites (about 4%) were located out of the ratio range of 0.5-2 (Figure 3A). Obviously, as the sites were reproducibly quantified by multiple glycopeptides, the quantitative results filtered by RSD for the first group were more reliable. Though the N-glycosites quantified from only one digestion strategy were less reliable (group two), over 95% of the quantified ratios located within the range of 0.5-2 indicated the workflow has good performance in quantification. If the confidence of quantification is defined as the percentage of correctly quantified ratios (the ratios quantified in the range of 0.5-2) among all quantified ratios, then the confidence for above two classes of quantified ratios were 99.9% and 95% respectively.

### Differential analysis of the N-glycoproteome of normal human liver tissues and human liver tissues with hepatocellular carcinoma

**Figure 4:**
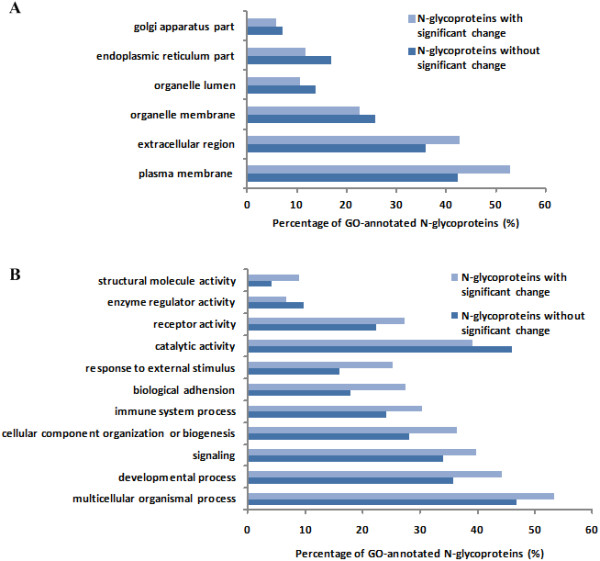
**The bar graph showing the different subcategories of GO-annotated proteins for N-glycoproteins with and without significant change between the HCC and normal human liver tissues. (A)** cellular components, **(B)** biological processes and molecular functions. Only the classes with the difference in the percentages over 10% for the two types of N-glycoproteins were given.

This workflow was then applied to differential analysis of the N-glycoproteome of HCC and normal human liver tissues. Because of the undersampling problem of mass spectrometry (MS), the pooled labeled glycopeptides from each digestion strategy were analyzed by 2D nanoLC-MS/MS three times (three replicate runs) to achieve as much information as possible for the sample. An average of 1,063 N-glycosites (RSD = 3.7%, n = 3) was quantified by trypsin digestion, 1,046 N-glycosites (RSD = 1.6%, n = 3) was quantified by trypsin & Glu-C digestion, and 301 N-glycosites (RSD = 8.1%, n = 3) was quantified by chymotrypsin digestion. The low RSD for the number of quantified N-glycosites among three replicates indicated the good reproducibility of the workflow. Totally, 2,329 N-glycosites corresponding 1,052 N-glycoproteins were successfully quantified in the combined digestion results from three digestion strategies, of which 754 N-glycosites (32.4%) were additionally quantified by trypsin & Glu-C digestion and chymotrypsin digestion (Figure 2B, Additional file 3: Table S2). First of all, the results were filtered by the RSD criterion (RSD <50%) in three replicates analyses of each digestion strategy to control the quantification accuracy of technical replicates. Then the filtered results of 2,101 N-glycosites were classified with the method described above. 858 N-glycosites were accurately quantified with RSD <50% for the ratios obtained from more than one digestion strategy. 275 of 858 N-glycosites were located out of the ratio range of 0.5-2 (heavy/light ratio), which was considered to be of significant expression change with more than 99% confidence. 1,104 N-glycosites were only quantified from only one digestion strategy, 428 of which with the ratio >2 or <0.5. These sites were significantly changed in abundance with more than 95% confidence (Figure 3B).Only the 858 N-glycosites with more than 99% confidence were subjected to further investigation. The N-glycoproteins containing the N-glycosites with or without significant changes were all analyzed by GoMiner to obtain a comparative view of cellular component, biological process and molecular function. For the 583 N-glycosites on 369 N-glycoproteins determined without significant change between HCC and normal human liver tissues, 328 proteins of which were found to be gene ontology (GO)-annotated proteins. And for the 275 N-glycosites on 215 N-glycoproteins determined with significant change, 190 proteins of which were with GO annotation. Comparing the N-glycoproteins with significant change and that without significant change, the percentage of GO-annotated proteins related with the intracellular organelles in the process of glycoprotein synthesis such as golgi apparatus and endoplasmic reticulum was decreased by more than 10% (Figure 4A). Meanwhile, for the N-glycoproteins with subcellular annotation of “plasma membrane” and “extracellular region”, both the proportions were increased by more than 10% in the part of N-glycoproteins with significant change. It can be inferred that the enhanced tranport of N-glycoproteins from the intracellular organelles to extracellular matrix may play a vital role in the development of HCC. The featured biological processes involved with membrane proteins and secreted proteins, such as “multicellular organismal process”, “developmental process”, “signaling”, “cellular component organization or biogenesis”, “immune system process”, “biological adhension” and “response to external stimulus”, were remarkably overexpressed in the significant changed N-glycoproteins part versus the no-change part. Additionally, the molecular functions characteristic for N-glycoproteins, e.g. “catalytic activity”, “receptor activity”, “enzyme regulator activity” and “structural molecule activity”, were also represented differently by more than 10% between the N-glycoproteins part with and without significant change (Figure 4B). Therefore, the differential expressed N-glycoproteins related with these biological processes and molecular functions mentioned above may contribute greatly to the progression of HCC, but further investigations were still needed.

## Discussion

Glycoproteins with different linkage of various glycans to Asparagine get involved in multiple critical biological processes, such as cell adhesion, cell migration, signal transduction, receptor activation and so on [26]. Dysregulation of glycoprotein, either on the glycan part or the polypeptide part, is associated with many types of diseases, e.g. cancer, diabetes mellitus, congenital disorder, arthritis, inflammation and so on [26-28]. Although the high-throughput identification of N-glycosites has been extensively carried out on varieties of biological samples, such as mouse tissues [29], human serum/plasma [6,30], seven major model organisms [31], the large scale quantitative analysis of N-glycosites were still lacked and urgently needed. So we designed this integrated workflow incorporating multiple protease digestion and solid phase based labeling method to investigate the differential expression in the N-glycoproteome of HCC and normal human liver tissues.

Nonenzymatic deamidation of glutamine and asparagine occurs spontaneously on proteins and peptides both in vivo and in vitro. For large-scale proteomics analysis, the determination of N-glycosites generally depends on the detection of the deamidation of asparagine in the peptides containing the consensus motif N-X-S/T (X can be any amino acid except proline). Because the nonenzymatic deamidation can also occur on the asparagine in the consensus sequence which is not actually occupied by a glycan, this may cause false positive identification of N-glycosites [32]. Hao et al. ascertained that nonenzymatic asparagines deamidation occurred to some extent on 4-9% of the peptides obtained by an electrostatic repulsion-hydrophilic interaction chromatography (ERLIC) – reverse phase (RP) chromatography, resulting in the false positive identifications of many N-glycosites [33]. Zielinska et al. [29] have used H_2_^18^O to differentiate native deamidation sites from those occurring during sample preparation, but their approach cannot distinguish the nonenzymatic deamidation that occurs in the consensus sequence during the PNGase F treatment from the real N-glycosites. Compared with above mentioned methods, our workflow was based on solid phase. Sequences with nonenzymatic deamidation that occurs during sample preparation can be cleaned away from the surface of hydrazide resins by rigorous and repeated washings. The only factor in our workflow which may cause false positive identifications was the incubation with PNGase F in the 37°C overnight. To estimate the false positive identifications in our workflow, a negative control with the same experimental procedures but with the omission of PNGase F addition was carried out. Less than 1% of the total quantified N-glycosites in the consensus sequences were also quantified in the negative control (data not shown). Consequently, the false positive identifications caused by nonenzymatic deamidation can be neglected in our workflow.

For large scale proteomic analysis, it would cost several days of precious MS times to analyze a couple of samples [15,34]. It is impractical to analyze large number of samples via large scale proteomics approaches. According to the process flow for the development of novel protein biomarker candidates described by Rifai et al. [35], a simplified, binary comparison between diseased and normal tissues was usually performed by proteomics methods based on MS for the discovery of biomarker candidates. So in practice, different samples were pooled together to reduce the biological variation [29,36-38]. For further analysis, the molecules screened out by large scale proteomics should be individually validated in each case among a large cohort of samples which may involve the environmental, genetic, biological and stochastic variation [39,40]. Our integrated workflow was aimed to provide lists of potential biomarker candidates by differentially analyzing the N-glycoproteome between HCC and normal human liver tissues in a high-throughput manner.

Among the total of 455 N-glycoproteins which was determined with significant change between HCC and normal human liver tissues in our experiment, 116 N-glycosylated molecules are found to be related with various types of human disease in the biomarker filter analysis by Ingenuity Pathway Analysis (IPA), such as cancer, cardiovascular disease, immunological disease and so on. Furthermore, some of the 90 N-glycosylated molecules involved in the human liver diseases are previously reported to be associated with liver cancer (Additional file 4: Table S3). For example, decorin (DCN), a secreted small leucine-rich proteoglycan, has three N-glycosites annotated by Swiss-Prot (N211, N262, N303), two of which were determined with decreased level in HCC versus normal liver tissues (N262 0.65, N303 0.47). Our result was consistent with the findings of Baghy et al., in which the idea that decorin acts as a secreted tumor repressor during hepatocarcinogenesis by hindering the action of another receptor tyrosine kinase, such as the PDGFRα, was supported and DCN was suggested be a novel therapeutic agent in the battle against liver cancer [41]. GPNMB, with N-glycosites of N128 quantified with increased level in HCC in our study, was also demonstrated with significantly enhanced expression level in HCC compared with adjacent normal liver tissues by using histochemical method in the study of Tian et al. [42]. Subramaniam et al. have viewed specific suppression of IGFBP3 expression in primary human HCC tissues compared to adjacent histologically normal tissues [43], while the significant down-regulation of IGFBP3 (N116 0.4) were also observed in our experiment. Besides, many N-glycosyalted molecules which may also be involved in other types of cancer were also detected in our results, e.g. elevated ALCAM shedding in colorectal cancer was correlated with poor patient outcome [44], BMPR2 participated in the regulation of endoglin-mediated suppression of prostate cancer invasion [45], CD36 was demonstrated to be an important index for predicting the occurrence and development of radiation pneumonitis and evaluating the curative effect [46], etc.

## Conclusions

Quantitative proteome analysis is a valuable tool to screen the disease-specific biomarkers. In this work, an integrated glycoproteomics workflow was applied to differential analysis of N-glycoproteome between HCC and normal human liver tissues. Multiple protease digestion was also integrated with the solid phase based labeling to increase the coverage of human liver N-glycoproteome analysis and the confidence of the quantified N-glycopeptides. A total of 2,329 N-glycosites matched with the motif N-X-S/T (X can be any amino acid except proline) on 1,052 N-glycoproteins were successfully quantified. Consequently, our integrated workflow will also perform effectively when targeting general population screening of differential N-glycoproteins in HCCs, and provides a valuable public dataset for further verification and validation of potential biomarker candidates of HCC.

## Methods

### Protein extraction

The HCC and normal human liver tissues were provided by Second Affiliated Hospital of Dalian Medical University (Dalian, China). The normal human liver tissues were the noncancerous liver tissues ≥ 2 cm outside the hepatic cancer nodules removed by surgical operation from patients. The noncancerous liver tissue has been verified by histopathological examination which excluded the presence of invading or microscopic metastatic cancer cells. The HCC tissues were obtained from the HCC patients of advanced stage by surgical operation. The utilization of human tissues was complied with guidelines of Ethics Committee of the Hospital.

The isolated human liver tissue was cut into pieces at first and washed several times with PBS buffer to remove the remaining blood. For large scale proteomic analysis, the noncancerous liver tissues from five patients with hepatic cancer were pooled together to reduce the biological variation [29,36-38], and so were the HCC tissues. Then the liver tissue was placed in an ice-cold homogenization buffer I (10 mM HEPES, 1.5 mM MgCl_2_, 5 mM KCl, 0.1 mM EDTA, 2% protease inhibitor cocktail (pH = 7.4)), followed by homogenization using an IKA Ultra Turbax blender. After that, the tissue was transferred into a Potter-Elvejhem homogenizer with a Teflon piston and homogenized for a second time on the ice. The supernatant was collected after centrifugation of the homogenates at 1,000 g for 5 min to pellet the nuclei and debris. Then 5 volumes of buffer II (0.1 M Tris–HCl, 4% SDS, 1% Triton, pH = 7.4) was added to the sample for sonication using an ultrasonic cell disrupter (3 s with 3 s intervals for 180 times at 400 W) and centrifuged at 20,000 g for 15 min. The supernatant was collected and the concentration of proteins was measured by BCA assay.

### Oxidation of glycan and enrichment of N-glycoproteins

Three batches of 2 mg human liver extract were pretreated in parallel. Firstly, the samples were desalted with Zeba spin desalting column (Thermo Scientific, USA) after incubation in boiling water for 10 min. Then the desalted solutions were resuspended with oxidation buffer (150 mM NaCl + 100 mM NaAc + 2% SDS + 2% Triton, pH 5.5). Sodium periodate was added into the solutions to oxidize the glycan in the dark at room temperature for 1 h. Finally, the oxidized samples were added into the prewashed hydrazide resins (Bio-Rad, USA) after quenching the oxidation reaction with sodium thiosulfate. The coupling reaction was performed with gentle shaking at room temperature overnight.

### Digestion of the captured N-glycoproteins on hydrazide resins with multiple proteases

After the removal of supernatant, the resins with captured N-glycoproteins were diluted with 50 mM Tris + 1% SDS + 1% Triton (pH = 8). The glycoproteins on the resins were reduced with 20 mM dithiothreitol at 37°C for 2 h and carboxyamidomethylated with 40 mM iodoacetamide at room temperature for 40 min in the dark. Then 100 mM NH_4_HCO_3_, 80% ACN and 100 mM NH_4_HCO_3_ were sequentially added to wash the resins to remove non-specifically bound proteins. Trypsin, trypsin & Glu-C, and chymotrypsin were chose as proteases to digest the three batches of captured glycoproteins on the resins respectively. For trypsin & Glu-C digestion, the two proteases were added into the sample together. The experimental procedures with each protease were all the same but different with the quantity of proteases added.

After the last wash of 100 mM NH_4_HCO_3_, each protease was added to digest the captured glycoproteins. The digest conditions of each protease were described as follows: Trypsin (Sigma-Aldrich, USA) was added with a weight ratio of trypsin to protein at 1/25 and incubated at 37°C overnight; Trypsin & Glu-C were added with a weight ratio of trypsin to protein at 1/25 and Glu-C (Roche, Germany) to protein at 1/20 and incubated at 37°C overnight; Chymotrypsin (Sigma-Aldrich, USA) was added with a weight ratio of chymotrypsin to protein at 1/10 and incubated at 37°C overnight.

### Dimethyl labeling the captured N-glycopeptides on the hydrazide resin and releasing N-glycopeptides from hydrazide resins

After incubation with the selected protease overnight, 1.5 M NaCl, 80% ACN and 100 mM NH_4_HCO_3_ were added sequentially to wash away the digested peptides from resins. As described in our previous work [12], 400 μL 100 mM TEAB was added to the hydrazide resins, followed by the addition of 32 μL 4% CH_2_O/CD_2_O to the sample to be light or heavy labeled respectively and the additional addition of 32 μL 0.6 M NaBH_3_CN to both samples. The dimethyl labeling reaction was carried out at room temperature for 2 h with gentle shaking. After rinsing the hydrazide resins twice with deionized water to remove the remaining dimethyl labeling reagents, the N-glycopeptides were released by adding 1,000 unit PNGase F (New England Biolabs, USA) in 10 mM NH_4_HCO_3_ to the resins and incubating at 37°C overnight with gentle shaking. The released deglycosylated peptides were carefully collected by gentle centrifugation.

### Mass spectrometry data acquisition

The enriched N-glycopeptides were analyzed by 2D nanoLC-MS/MS system [17] on LTQ-Orbitrap Velos (Thermo, San Jose, CA, USA) and one tenth of the labeled N-glycopeptides were analyzed each time. The LTQ-Orbitrap Velos system was equipped with an Accela 600 HPLC (Thermo, San Jose, CA, USA) involving a 7 cm phosphate monolithic trap column and a 12 cm C18 capillary analysis column with spray emitter. The SCX column was prepared by the same method as described by Wang et al. [47] and separation column was packed with C18 AQ beads (3 μm, 120 Å). Formic acid water solution (Buffer A) and pure acetonitrile (ACN) with 0.1% formic acid (Buffer B) were used for the generation of linear gradient for RPLC separation. The peptide sample was first loaded onto the monolithic trap, then a series stepwise elution with salt concentrations of 100, 200, 300, 400, 500 and 1,000 mM NH_4_Ac was used to gradually elute peptides from the phosphate monolithic column onto the C18 analytical column. After each salt elution, binary separation gradient from 5% to 35% in 120 min with a flow rate of ∼ 300 nL/min was applied to separate peptides prior to MS detection.

A spray voltage of 2.2 kV was applied between the spray tip and the MS interface. The temperature of the ion transfer capillary was set as 250°C. The mass spectrometer was set that one full MS scan was followed by 20 MS/MS scans on the 20 most intense ions by collision induced dissociation (CID). 300 was set as the minimum signal threshold for MS/MS scan. The normalized collision energy was set at 35.0%, and the activation time was 10 ms. The mass resolution was set at 60 000 for full MS. The dynamic exclusion was set as follows: repeat count, 1; duration, 30 s; exclusion list size, 500; exclusion duration, 90 s. The scan range was set from m/z 400 to 2,000. System control and data collection were carried out by Xcalibur software version 2.1.

### Protein identification and quantification

All the RAW files collected by Xcalibur 2.1 were searched by MaxQuant version 1.2.2.5 against a composite database including original and reversed human protein database of International Protein Index (IPI human v3.80 fasta, including 86,719 entries, ftp://ftp.ebi.ac.uk/pub/databases/IPI). The parameters were set as follows: proteases, trypsin, 2 missed cleavages (trypsin & Glu-C, 4 missed cleavages or chymotrypsin, 4 missed cleavages); static modification, Cysteine Carboxamidomethylation; variable modification, oxidation of methionine and deamidation of glutamine/asparagine; mass tolerance, 20 ppm for parent ions and 0.5 Da for fragment ions. For the quantitative analysis of the results, peptide N termini and the side chain of lysine residues were set with light (+28.0313 Da) and heavy dimethylation labels (+32.0564 Da). The false detection rate (FDR) was determined by equation of FDR = [2 × FP / (FP + TP)] × 100%, where TP (true positive) is the number of peptides that were identified based on sequences in the forward database component and FP (false positive) is the number of peptides that were identified based on sequences in the reverse database component. The FDR values for peptide identifications were controlled less than 1%. GoMiner, the software which can make the biological interpretation in the context of the Gene Ontology [48], was applied to classify the cellular component, biological process and molecular function of the quantified N-glycoproteins between HCC and normal human liver tissues with the newly updated database (version_go_201202).

## Competing interests

The authors declare that they have no competing interests.

## Authors’ contributions

HZ initiated this study, HZ and MY designed experiments; ZS performed most of the experiments; ZS, FW and KC analyzed data; ZZ and BX participated in glycoprotein enrichment and dimethyl labeling; DS and LW performed tissue collection and histopathological examination; ZS, FW, MY and HZ wrote the manuscript. All authors read and approved the final manuscript.

## Supplementary Material

Additional file 1: Table S1 The detailed information about the N-glycoproteins and N-glycosites quantified from normal human liver tissues. Click here for file

Additional file 2: Figure S1 The distribution of the percentage of quantified N-glycosites within the ratio range of 0.5-2 in three ranges of RSD value (>50%, 30%-50%, 20%-30%) (A), and the number of quantified N-glycosites filtered with different RSD values (<20%, <30% or <50%) (B), in the results of the evaluation experiment. Click here for file

Additional file 3: Table S2 The detailed information about the N-glycoproteins and N-glycosites quantified from HCC and normal human liver tissues. Click here for file

Additional file 4: Table S3 The detailed information about quantified N-glycoproteins and N-glycosites involved in human liver diseases. Click here for file
