# Supplementary figures and images for: Differential analysis of N-glycoproteome between hepatocellular carcinoma and normal human liver tissues by combination of multiple protease digestion and solid phase based labeling
Source: Clin Proteomics. 2014 Jul 1;11(1):26. doi: 10.1186/1559-0275-11-26 (PMC4112855; doi:10.1186/1559-0275-11-26)

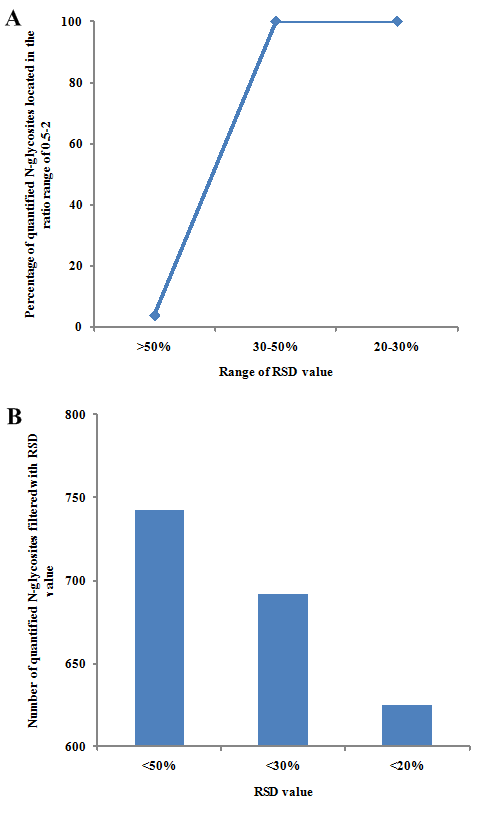

Supplement: Additional file 2: Figure S1 — The distribution of the percentage of quantified N-glycosites within the ratio range of 0.5-2 in three ranges of RSD value (>50%, 30%-50%, 20%-30%) (A), and the number of quantified N-glycosites filtered with different RSD values (<20%, <30% or <50%) (B), in the results of the evaluation experiment. [file 1559-0275-11-26-S2.tiff]
